# MerTK inhibition is a novel therapeutic approach for glioblastoma multiforme

**DOI:** 10.18632/oncotarget.1793

**Published:** 2014-03-12

**Authors:** Kristina H. Knubel, Ben M. Pernu, Alexandra Sufit, Sarah Nelson, Angela M. Pierce, Amy K. Keating

**Affiliations:** ^1^ Department of Pediatrics, University of Colorado School of Medicine, Aurora, CO, USA

**Keywords:** MerTK, Axl, glioma, Foretinib, intracranial model

## Abstract

Glioblastoma is an aggressive tumor that occurs in both adult and pediatric patients and is known for its invasive quality and high rate of recurrence. Current therapies for glioblastoma result in high morbidity and dismal outcomes. The TAM subfamily of receptor tyrosine kinases includes Tyro3, Axl, and MerTK. Axl and MerTK exhibit little to no expression in normal brain but are highly expressed in glioblastoma and contribute to the critical malignant phenotypes of survival, chemosensitivity and migration. We have found that Foretinib, a RTK inhibitor currently in clinical trial, inhibited phosphorylation of TAM receptors, with highest efficacy against MerTK, and blocked downstream activation of Akt and Erk in adult and pediatric glioblastoma cell lines, findings that are previously unreported. Survival, proliferation, migration, and collagen invasion were hindered *in vitro*. Foretinib treatment *in vivo* abolished MerTK phosphorylation and reduced tumor growth 3-4 fold in a subcutaneous mouse model. MerTK targeted shRNA completely prevented intracranial and subcutaneous glioma growth further delineating the impact of MerTK inhibition on glioblastoma. Our findings provide additional target validation for MerTK inhibition in glioblastoma and demonstrate that robust MerTK inhibition can be achieved with the multi-kinase inhibitor Foretinib as an innovative and translational therapeutic approach to glioblastoma.

## INTRODUCTION

The TAM subfamily of receptor tyrosine kinases (RTKs) comprised of Tyro3, Axl and MerTK, have high structural homology and share a common ligand Gas6. The hallmarks of this family include two fibronectin type III domains, two immunoglobulin-like domains and a conserved sequence within the kinase domain [1]. TAM RTKs have a tissue-specific expression pattern, with higher expression in the immune, nervous and reproductive systems [2-5]. TAM RTKs have well described normal physiologic functions including phagocytosis, synaptic pruning, immune-regulation and sexual maturation [6-9]. Notably, MerTK activation leads to known anti-apoptotic survival signal, mediating through the MAPK and PI3K pathways both in normal conditions as well as situations of oxidative stress [10]. Aberrant expression of Axl and MerTK has been described in multiple cancers, including glioblastoma, and in some cases Axl or MerTK overexpression or co-expression has been correlated with poor clinical outcomes [11-14]. Interestingly, there is little to no expression of either Axl or MerTK in normal CNS tissues [9, 13, 15], making targeting TAM RTKs an attractive novel therapeutic strategy for glioblastoma.

Glioblastoma is characterized by insistent growth, high rate of migration and relentless recurrence, with 30,000 new patients diagnosed annually [16]. It is one of the most common solid tumors in both pediatric and young adult patients and remains relatively incurable [17]. The standard backbone therapy consists of surgical resection and radiotherapy results in a median survival time of 12 months, while the addition of temozolimide increases the median survival time to 16 months [18, 19]. In addition to being largely non-curative, these therapies are devastating in a developing pediatric brain with profound consequences on neuro-cognition, growth, and endocrine function [20]. Targeted therapies against RTKs, such as EGFR (Gefitinib), have thus far not produced a significant survival advantage over standard therapy. This is likely due to the large amount of molecular heterogeneity found between individual patient tumors; only a minority of patients has overexpression of the RTK being targeted. Additionally, many tumors have RTKs that are mutated and consequently are not susceptible to targeted inhibition. In contrast, we have previously found that there is high aberrant expression of either MerTK or Axl in all grades of astrocytic tumors tested, and that co-expression of MerTK and Axl was present in all high grade gliomas and glioblastoma patient samples [15], additionally sequence analysis found no receptor mutations (unpublished data). We have also shown that knockdown of MerTK and Axl by stable shRNA resulted *in vitro* in increased apoptosis, decreased cell proliferation, and improved sensitivity to temozolimide, carboplatin, and vincristine [15]. Inhibition of MerTK was found to greatly reduce glioblastoma migration and alter cellular morphology [21]. From this data we sought to study the effects of MerTK, and perhaps Axl and Tyro3, inhibition *in vivo* utilizing a multi-kinase translational inhibitor which effectively blocks activation of these receptors.

Foretinib is a kinase inhibitor whose best known targets are c-Met and VEGFR2/KDR [22]. Currently, there are a number of phase II clinical trials in progress using Foretinib to treat breast, liver and gastric cancers, papillary renal cell carcinoma, and squamous cell head and neck cancer [23-28]. Although Foretinib was designed as a cMet/VEGFR inhibitor, it has reported activity against Axl at lower concentrations than cMet [28], however the ability to target MerTK and Tyro3 has not previously been described.

With this study, we establish for the first time that Foretinib inhibits all of the TAM family members, and has highest potency against MerTK in the glioblastoma cells studied. We demonstrate that with Foretinib therapy we can replicate the *in vitro* inhibition of survival and migration of glioblastoma seen following TAM RTK genetic inhibition, and we validate the therapeutic potential of TAM inhibition in *in vivo* models and the necessity of MerTK for glioblastoma tumor growth.

## RESULTS

### Foretinib inhibits the activation of TAM family receptors in glioblastoma cells

**Figure 1:**
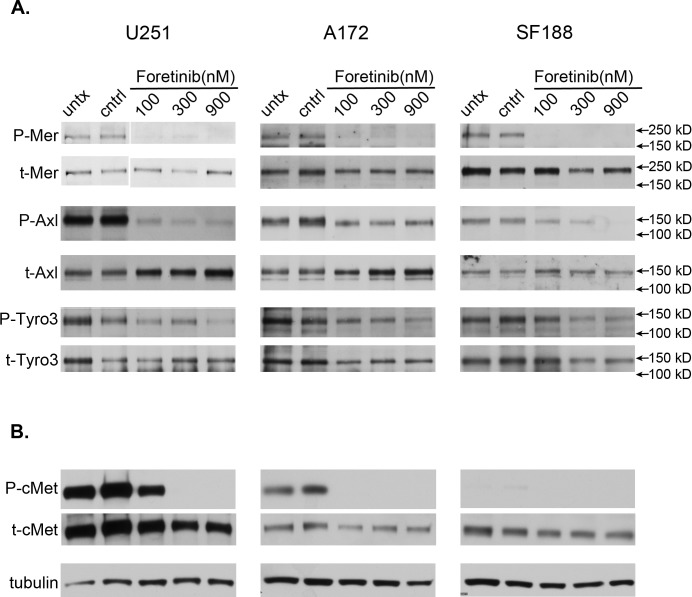
Foretinib treatment effectively targets the activation of TAM RTK family members a. U251 (left), A172 (middle) and SF188 (right) glioblastoma cells were left untreated (untx), treated with vehicle only (cntrl), or with Foretinib at increasing concentrations. Cells were harvested at 1 hr in the presence of pervanadate and whole cell lysates were prepared and immunoprecipitated with antibodies against the TAM family members, and resolved with SDS PAGE. Samples were blotted for the activated phospho- form (P-TAM) and stripped and re-probed for the total form (t-TAM). b. Similar cell preparations were collected without pervanadate treatment, resolved with SDS PAGE directly and immunoblotted for the known target of Foretinib, activated c-Met (P-cMet) and total cMet (t-cMet) in U251 (left), A172 (middle) and SF188 (right) cells. Tubulin was used as a loading control. Blots are representative of three independent experiments.

Inhibition of TAM family members may be a novel therapeutic approach to treat glioblastoma; therefore we evaluated the phosphorylation state of the TAM family members in response to Foretinib treatment in the adult glioblastoma cell lines, U251 and A172, and the pediatric glioblastoma cell line SF188. Foretinib treatment at the lowest concentration tested, 100 nM, completely inhibited the phosphorylation of MerTK in all three cell lines (Figure 1A). Similarly, phospho-Axl was inhibited considerably at all concentrations tested in the U251 cell line, while in the SF188 line inhibition followed a concentration dependent trend. The A172 cell line showed partial inhibition of Axl activation at 100nM that did not increase with increasing doses in the range tested. The phosphorylation of Tyro3 in the U251 cell line was inhibited at 900 nM Foretinib, however, conclusions of the level of activation/inhibition of Tyro3 regarding the other two cell lines cannot be accurately assessed from this data because total levels of Tyro3 changed as well. Most notably, Foretinib at 100 nM did not inhibit the phosphorylation of cMet in U251 cells (Figure 1B left panel). SF188 cells do not seem to have appreciable activation of cMet even at baseline and likely does not have a large role in the downstream signals nor the functional phenotypes of this cell line despite having similar levels of total cMet as the U251 and A172 cells (Figure 1B right panel). MerTK is more highly expressed in SF188 cells compared to U251 cells, whereas the opposite is true for Axl (data not shown). We have shown that the lowest concentration of Foretinib (100 nM) used in this study always inhibited the activity of MerTK, whereas the higher concentrations of Foretinib (300-900 nM) inhibited the activity of Axl, Tyro3. From this we conclude that activation of TAM family members, and specifically MerTK, are successfully blocked in glioblastoma at concentrations lower than 1mM.

### Foretinib inhibits activation of oncogenic signaling pathways in glioblastoma

**Figure 2:**
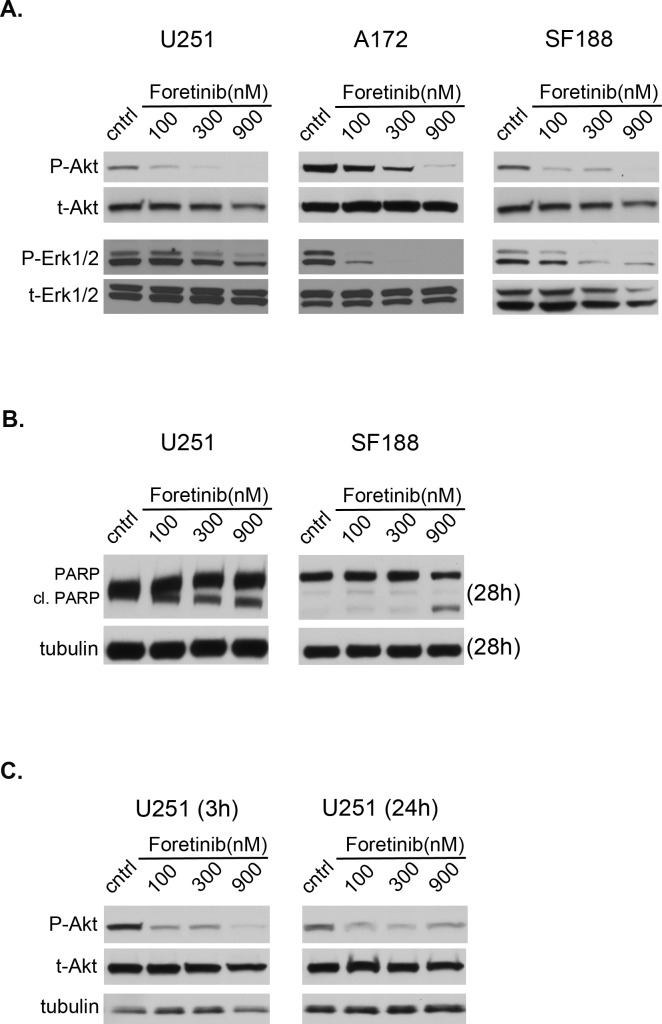
Foretinib inhibits activation of pertinent oncogenic signaling pathways a. U251 (left), A172 (middle) and SF188 (right) cells were treated with vehicle only (cntrl) or Foretinib at increasing concentrations in full serum media. Cells were harvested at 1 hr and whole cell lysates were prepared and resolved by SDS PAGE. Samples were blotted for the phospho- form and re-probed for the respective total form of Akt and ERK1/2. b. Cells in full serum media were treated as described, harvested at 28 hrs and probed for apoptotic indicators, PARP and PARP cleavage (cl. PARP). c. Whole cell lysates were also harvested after treatment as described from U251 cells after continuous treatment of foretinib for 3 and 24 hrs to assess the longevity of downstream pathway inhibition. Tubulin was used as a loading control. Blots are representative of three independent experiments.

Next, we investigated the involvement of downstream kinase signaling pathways in response to Foretinib. Foretinib treatment decreased Akt phosphorylation in a concentration dependent manner in all three cell lines at 1 h (Figure 2A). Akt activity was still decreased at the 3 and 24 h time point (Figure 2C). Similar results were seen with phospho-ERK (Figure 2A), but it had no effect on p38 (data not shown). Moreover, indication of the apoptotic effect by Foretinib treatment was evidenced by PARP cleavage assayed at 28 h post-treatment in full serum media (Figure 2B). Therefore, Foretinib down-regulates pertinent oncogenic signaling pathways known to be involved in survival, growth, and apoptosis regulation.

### Foretinib decreases short- and long-term cellular survival in a dose dependent manner

**Figure 3:**
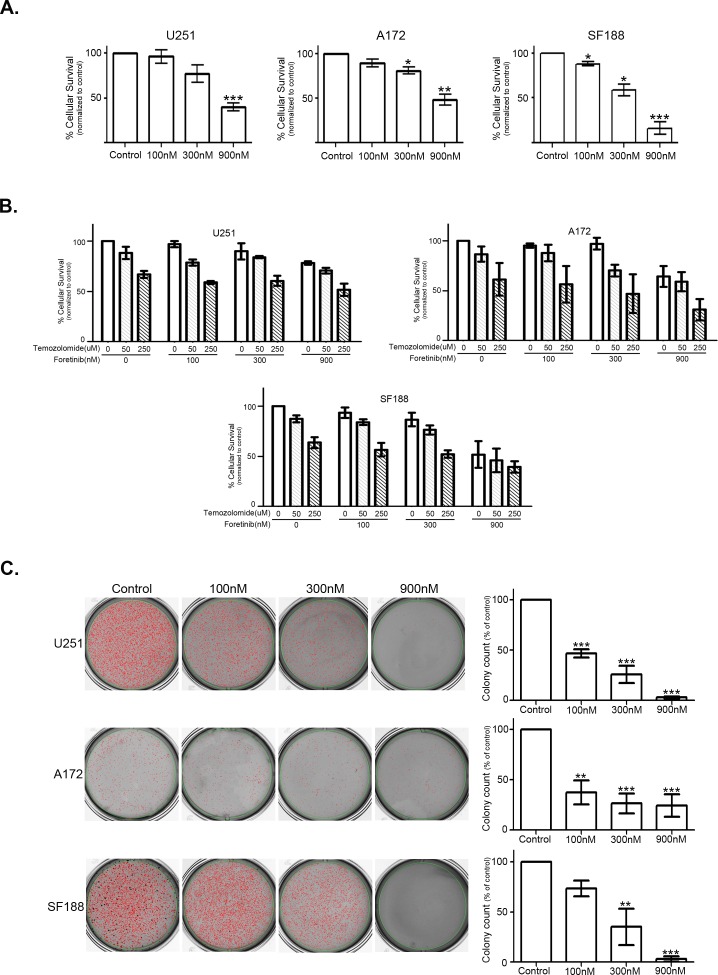
Inhibition of TAM RTKs with Foretinib decreases short- and long-term cellular survival and proliferation in a dose dependent manner a. U251 (left), A172 (middle) and SF188 (right) cells were plated with serum free conditions in quadruplicate in 96 well plates and treated with vehicle only (Control) or Foretinib at the indicated doses. Cell number was assessed with a MTT assay at 48 hr and normalized to control. The experiment was independently repeated three times and absorbance means and standard error of the mean were calculated. b. U251, A172 and SF188 cells were plated in full serum in quadruplicate in 96 well plates and treated with Foretinib and temozolomide at the indicated doses. Cell number was assessed with an MTT assay and measurement of absorbance at 48 hr. The experiment was independently repeated three times and absorbance means and standard error of the mean were calculated and normalized to vehicle only treatment (Foretinib 0 nM, temozolomide 0 uM). A mixed model repeated measurements ANOVA with a Bonferroni post-test adjustment was done to compare treatments alone and the various combinations to the control vehicle only treated and the single agent treatments. c. U251 (top), A172 (middle) and SF188 (bottom) cells were plated in triplicate in soft agar and treated with vehicle only (Control) or Foretinib at the indicated doses 2 times a week. Colonies were stained and counted at 3 weeks (n=3) and representative pictures were taken. Colony count means were normalized to vehicle control and standard error of the mean was calculated. For both short- and long-term analysis repeated measurements ANOVA with a Dunnet's multiple comparison test (*P<0.05; **P<0.01; ***P<0.001) was done to compare treatment to control.

Foretinib robustly inhibits TAM signals, and specifically MerTK, which are known to activate anti-apoptotic and pro-proliferative signals [29-31]. To investigate short- and long-term survival following treatment with Foretinib, we used two different approaches: measurement of metabolic activity (MTT reduction) at 48 hrs in serum-free media and long term proliferation in a soft agar non-adherent growth assay. To highlight short-term survival following Foretinib treatment, we used serum-free culture conditions in order to assess cells in the absence of exogenous, over-abundant TAM ligands found in serum. Survival was markedly reduced by 50-83% with 900 nM Foretinib treatment in all three cell lines (Figure 3A). Survival of SF188 cells was reduced by the very low treatment concentration of 100 nM Foretinib, and was further inhibited in a dose dependent manner (Figure 3A). Current standard of care for glioblastoma relies upon local control with surgical resection and radiation therapy. Many adjuvant chemotherapy and biotherapy approaches have been attempted with little success; one exception has been the addition of temozolomide (TMZ) cytotoxic chemotherapy, which extended the median survival of patients by several months [18]. We sought to characterize cell survival with the addition of Foretinib to treatment with temozolomide, as this has become a standard therapy for many glioma patients and would likely form the backbone of a clinical trial. Using an *in vitro* analysis in serum replete media we found that Foretinib multi-kinase inhibition does not impede TMZ associated cell death (Figure 3B). The U251 and A172 cell lines are considered TMZ sensitive because the DNA repair protein O(6)-methylguanine-DNA methyltransferase (MGMT) is not expressed due to methylation of the promoter, while SF188 is considered TMZ resistant because it does express MGMT [32-34]. In all three cell lines the addition of Foretinib to TMZ treatment resulted in similar to decreased cell survival over TMZ alone, indicating that Foretinib has the potential to be effectively added to current standard therapy. Additional work needs to be done to evaluate timing of treatments and expand concentrations tested as well as introduce combination therapy into mouse models of the disease. To evaluate the long-term survival and proliferation of this solid tumor in an anchorage independent environment we analyzed soft agar colony formation at 3 weeks. Foretinib treatment significantly diminished long-term growth, with colony formation reduced to 37-74% of vehicle control with only 100 nM Foretinib treatment (Figure 3C). Notably, the SF188 cells showed quite significant inhibition of short- and long-term survival and proliferation, likely in large part due to TAM RTK inhibition as there is very little/no activation of cMet related signaling in this line.

### Foretinib abrogates migration and invasion of glioma cells in a dose dependent manner

**Figure 4:**
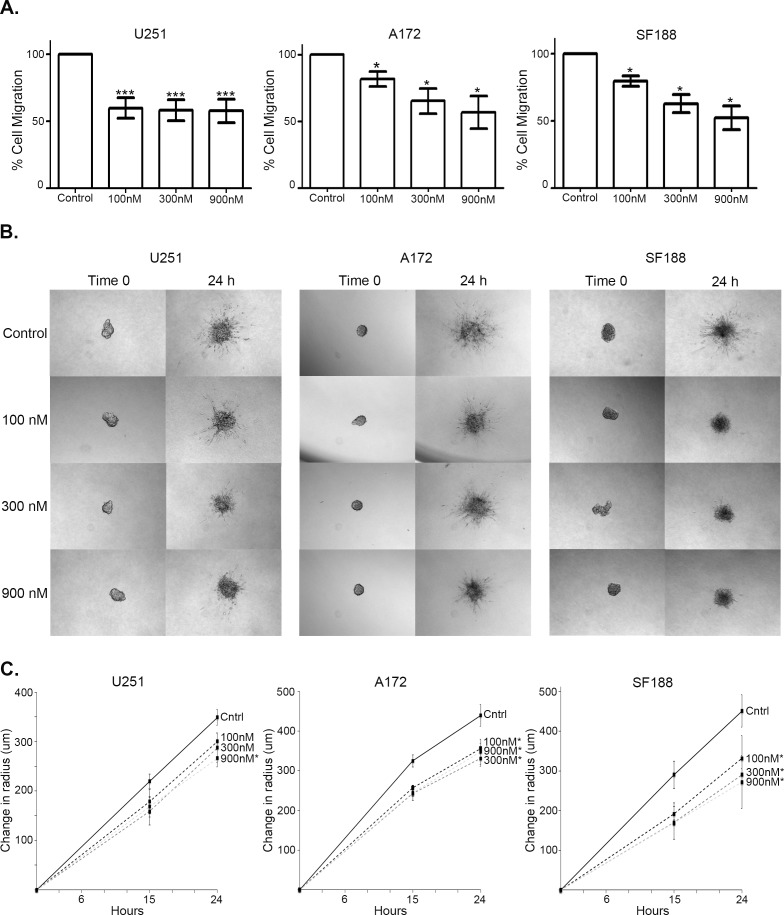
Foretinib hinders migration and invasion of glioma cells in a dose dependent manner a. U251 (left), A172 (middle) and SF188 (right) cells were plated in triplicate in xCELLigence CIM-Plates transwell cultures with 8 um pores and treated with vehicle only (Control) or Foretinib at the indicated doses and migration was measured as electrical impedence at the point of the transwell at 10 hr. Cell index means and standard deviations were calculated from three independent experiments and normalized to vehicle. A repeated measurements ANOVA with a Dunnet's multiple comparison test (*P<0.05; ***P<0.001) was done to compare treatment to control. b. Five neurospheres of both U251 (left), A172 (middle) and SF188 (right) were plated in a collagen matrix and imaged immediately (Time 0), treated with vehicle only (Control) of Foretinib at doses indicated and re-imaged at 15 hr (not shown) and 24 hr. Representative pictures were taken. c. Absolute maximal radius of each neurosphere was measured, and the mean and standard error of three independent experiments was graphed. A two-sided t-test was done at 24 hr to compare treatment to vehicle control (*P<0.05).

One of the major barriers to effective treatment of glioblastoma is its ability to migrate extensively out of the site of local control leading to relapse. We quantified cellular transwell migration in response to Foretinib using the xCELLigence real time cell analysis system. Foretinib treatment inhibited cell migration at the lowest concentration of 100nM in all three cell lines at 10 h post treatment, with U251 impeded to 59.8% of control, A172 to 80% of control, and SF188 to 81% of control (Figure 4A). Increasing doses of Foretinib further impeded migration in the A172 and SF188 cell lines, decreasing migration to 72-73% of control at 300 nM and 52-59% of control at 900 nM respectively (Figure 4A). This abrogation of transwell migration is not due to a change in cell number; we plated cells in parallel conditions to the transwell xCELLigence assay and then enumerated total cell number over the short timeframe of the experiment (10 h) and no changes in cell number were noted (Supplemental Figure S1).

In order to frame cellular movement more appropriately in the context of glioma, we chose a glioblastoma neurosphere model to perform a three dimensional collagen invasion assay. U251, SF188, and A172 cells were induced to form neurospheres [35], plated in collagen in 96 well plates, and treated with Foretinib. Migration of cells from the core of individual neurospheres were tracked and imaged at 15 and 24 h post treatment (Figure 4B and C). The absolute maximal radius of each neurosphere (core and furthest reaching cell) was measured and normalized to the radius at the time of plating (Time 0). Each data point represents the mean of three independent experiments where five individual neurospheres were matched and analyzed. TAM inhibition with Foretinib impeded the glioblastoma cellular invasion through a collagen matrix in all three cell lines both visually (Figure 4B) and quantitatively (Figure 4C). The lowest concentration of Foretinib impeded cell migration in A172 and SF188. In conclusion, Foretinib inhibits migration and invasion of glioblastoma *in vitro*, targeting two of the more aggressive features of clinical glioblastoma.

### Foretinib, a TAM RTK inhibitor, is an effective treatment for established subcutaneous human glioblastoma tumors and increases overall survival

**Figure 5:**
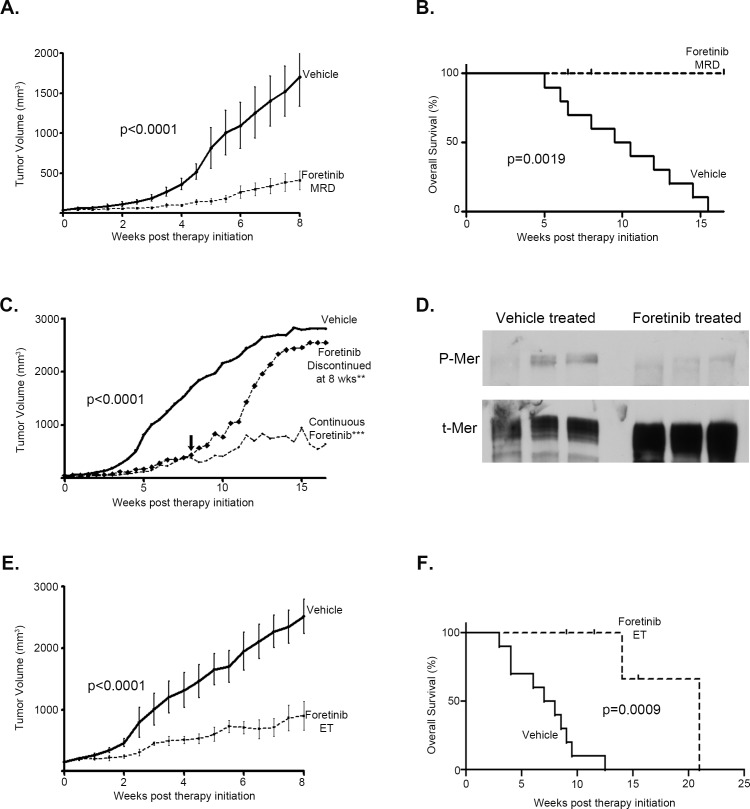
TAM RTK inhibition with Foretinib is an effective treatment for established subcutaneous human glioma tumors and increases overall survival U251 cells were subcutaneously implanted into the flanks of athymic nude mice and tumors were allowed to establish over 8 weeks. Mice were randomized into one of three groups: A control group received vehicle only, every other day (QOD) by oro-gastric (OG) administration for the entirety, another group initiated Foretinib treatment at 30 mg/kg QOD OG at 8 weeks post injection (Foretinib MRD), and the final group received the same Foretinib dosing regimen, initiated when the tumor reached 125 mm^3^ (Foretinib ET). Tumors were measured twice weekly by caliper and mean and standard error of the volumes were calculated. a. Vehicle (n=10) and Foretinib MRD (n=7) treated mice, which started therapy at 8 weeks post injection, had tumor volumes compared with a paired two-tailed t-test (p-values are provided) and b. a Kaplan-Meier analysis of survival (survival endpoint defined as sacrifice due to tumor size in excess of 2500 mm^3^ or morbidity due to tumor) with a log rank Mantel-Cox test statistical comparison (p-values are provided). c. A subset of the Foretinib MRD treated mice (n=4) had therapy discontinued at 8 weeks (down arrow) and tumor volumes were compared to vehicle and continuous Foretinib therapy (n=3). An ANOVA with Tukey's correction was used for statistical comparison to vehicle control (**P<0.01; ***P<0.001). d. Two tumors (vehicle (lanes 1-3) and foretinib treated (lanes 4-6)) were resected and immediately homogenized in lysis buffer with protease inhibitor with freshly prepared pervanadate (lanes 2 and 5) and without (lanes 3 and 6). Lanes 1 and 4 are samples that were not homogenized. Samples were resolved with SDS PAGE and then blotted for the activated phospho- form (P-Mer) and stripped and re-probed for the total form (t-Mer) to test for target inhibition. e. Vehicle (n=10) and Foretinib ET (n=5) treated mice, which started therapy when the tumor exceeded 125 mm^3^, had tumor volumes compared with a paired two-tailed t-test (p-values are provided) and f. a Kaplan-Meier analysis of survival with a log rank Mantel-Cox test comparison (p-values are provided).

Using U251 cells, we tested efficacy of Foretinib in an *in vivo* subcutaneous xenograft mouse model of human glioblastoma. Two different schedules were used: Foretinib was started at 8 weeks after cell injection, when the tumors were not yet in log-phase growth, mimicking a minimal residual disease (MRD) tumor burden that would remain following upfront surgical and radiation treatment; at this time the mean tumor volume was 51.5 mm^3^ (range 25.1 to 82.5 mm^3^) (Figure 5A,B), alternatively, animals were left untreated until the tumors were established (ET), defined by robust and rapid growth with a tumor volume of at least 125 mm^3^ (occurred between 10.5-15.5 weeks post injection) (Figure 5E,F). Animals were treated with vehicle as a control or with Foretinib at 30 mg/kg via orogastric administration every other day (QOD). We also explored the discontinuation of therapy following Foretinib therapy for 8 weeks, to further verify the critical role of Foretinib in inhibiting glioblastoma tumor growth (Figure 5C). Tumor volume was significantly decreased on both Foretinib treatment schedules compared with vehicle treatment regardless of whether Foretinib was started early or late in tumor development (Figure 5A and E). In the MRD treatment arm, tumors in the vehicle only control group (n=10) grew rapidly and surpassed a 750 mm^3^ median tumor volume at 6 weeks, while Foretinib MRD treated (n=7) mice never hit that threshold while on therapy (beyond 16.5 weeks). Tumors in the Foretinib MRD treated group were statistically smaller throughout the entirety of the treatment (Figure 5A), and at the end of the 8 weeks of therapy, the mean tumor volume in the vehicle control mice was 1702 mm^3^ (95% CI: 986-2420 mm^3^) while the Foretinib MRD treated mice it was 388 mm^3^ (95% CI: 117-659 mm^3^). The median survival for the vehicle only treated group was 10 weeks, while none of the mice on Foretinib MRD treatment reached tumor size endpoint requiring sacrifice (20 mm in any dimension, calculated volume of 2500 mm^3^, or morbidity due to tumor) while on therapy (Figure 5B). In the small subset of mice where treatment was discontinued at 8 weeks (n=4), the tumors quickly resumed logarithmic growth and the mice were euthanized between 4 and 7.5 weeks later due to reaching tumor size endpoint (Figure 5C), emphasizing the principal role of Foretinib inhibition on tumor growth. Foretinib very effectively targets MerTK activation *in vivo* as evidenced by the immunoblot showing significantly inhibited MerTK phosphorylation in the treated tumor as opposed to the vehicle treated tumor (Figure 5D). The blot is representative of a tumor removed from a mouse that was vehicle treated (left 3 lanes) or one that was Foretinib treated (right three lanes). Each of these tumors underwent protein extraction by three different methods: without homogenization (lanes 1 and 4, which resulted in unacceptable phospho-protein recovery), with homogenization in the presence of pervanadate (lanes 2 and 5), and with homogenization alone (lanes 3 and 6). The blot was stripped and re-probed for total MerTK (Figure 5D).

In the established tumor (ET) model analysis, the vehicle treated control group (n=10) had rapid tumor growth after reaching the 125 mm^3^ treatment initiation cut-off, and surpassed 1000 mm^3^ tumor volume at a median of 4.5 weeks later while in the Foretinib ET therapy group (n=5) tumor growth was suppressed through the entire treatment period even in these well-established tumors (Figure 5E). Tumors in the ET group treated with Foretinib were statistically smaller throughout the entirety of the treatment, and at the end of the 8 weeks of therapy the mean tumor volume in the vehicle control mice was 2721 mm^3^ (95% CI: 2181-3261 mm^3^) while the Foretinib treated mice it was 807 mm^3^ (95% CI: 351-1261 mm^3^). The median survival for vehicle treated mice was 7.5 weeks, while it was extended to 21 weeks in the Foretinib ET treated mice despite the rapidity of tumor growth in the ET group prior to the start of therapy (Figure 5F).

### MerTK signaling is required for glioblastoma growth *in vivo*

**Figure 6:**
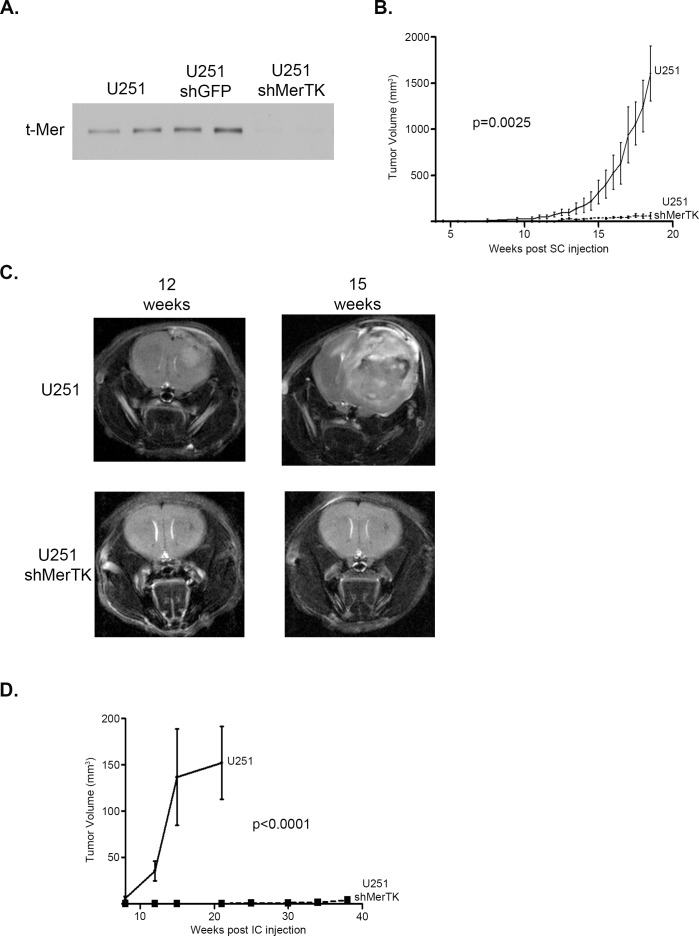
Specific MerTK inhibition with shRNA profoundly limits subcutaneous and intracranial human glioma growth a. Cell lines were developed from parental U251 to specifically knock down MerTK expression. Whole cell lysates were prepared and immunoblotted for total Mer expression (t-Mer). b. U251 cells or U251 cells transduced with shRNA against Mer (shMerTK) cells were subcutaneously implanted into the flanks of athymic nude mice and tumors were allowed to establish. The trial included three mice in both the control and experimental group. Tumors were measured twice weekly by caliper and mean and standard error of the volumes were calculated and means were statistically compared with a paired two-tailed t-test (p-values are provided). c. U251 cells or U251 cells transduced with shRNA against Mer (shMerTK) cells were injected orthotopically into the fronto-parietal region of the brain of athymic nude mice. MRI scans of mice were taken approximately every three weeks, with representative T2-weighted images of U251 control (top) and U251 shMerTK knockdown (bottom) injected mice from 12 (left) and 15 (right) weeks post injection shown here. d. Intracranial tumor volume was measured using Paravision Preclinical MRI Software. Two independent trials were completed, with each trial including three mice in both the control and experimental group, for a total analysis of six mice in each group. Mean and SEM were calculated and volumes were statistically compared with a paired two-tailed t-test (p-values are provided).

To illustrate the importance of MerTK in glioblastoma growth we developed genetically inhibited cell lines to use in *in vivo* modeling; U251 cells were transduced with shMerTK and sorted based on expression levels of MerTK following puromycin selection. Clonal cell lines were developed exhibiting stable and substantial MerTK knockdown (Figure 6A). U251 cells with MerTK knockdown or parental U251 cells were implanted subcutaneously. Tumor growth, by caliper measurement, in the control cells was rapid and pronounced with a final mean tumor volume of 1603.9 mm^3^ (range 1053.3 to 2068.6 mm^3^) at 18.5 weeks post-injection (p.i.), while growth was profoundly inhibited with shMerTK knockdown (Figure 6B), with a final mean tumor volume of 63.4 mm^3^ (range 20.7 to 134.5 mm^3^). Additionally, we pursued an orthotopic intracranial mouse model using these same cell lines, as the ultrastructure and microenvironment outside of the tumor would be most closely aligned with the human condition. Magnetic resonance imaging (MRI) done approximately every three weeks starting at 8 weeks p.i. demonstrated a profound difference in tumor growth, with all mice (n=6) in the control group having measureable tumor volumes by 12 weeks p.i. and significant tumor burden by 15 weeks p.i. (Figure 6C and 6D), while not a single shMerTK (n=6) had any tumor growth at that time. The median survival of the control mice was 15 weeks p.i., while all animals with shMerTK cells injected were alive and without significant disease burden at 38 weeks p.i. (Figure 6D). These results confirm the critical function of MerTK for *in vivo* glioblastoma growth. Furthermore, this study demonstrates that treatment with a translational tyrosine kinase inhibitor which effectively blocks MerTK activation yields similar effects *in vitro* and *in vivo* to selective MerTK knockdown via shRNA.

## DISCUSSION

Glioblastoma is a common and extremely aggressive malignancy that exhibits extraordinary invasive potential and a high level of recurrence; the outcomes are horrible and useful therapies are limited. TAM receptors, a subfamily in the receptor tyrosine kinase family, are a relevant target in glioblastoma because they are aberrantly expressed in glioblastoma and control both cellular survival and migration. We now demonstrate for the first time that the tyrosine kinase inhibitor Foretinib effectively inhibits TAM family members in glioblastoma, and most robustly MerTK, leading to an inhibition of the critical phenotypes of glioblastoma and a dramatic tumor response in *in vivo* mouse models of disease.

We believe that MerTK and Axl are appropriate targets for therapy against glioblastoma because they exhibit little to no expression at both the mRNA and protein level in the normal human brain but are upregulated in glioblastoma and are readily activated by the ubiquitously present Gas6 which is available in the CNS in the normal and the diseased state [36]. Tyro3 is more widely expressed in the normal brain [3, 37] but also, in our studies, shows the smallest response to Foretinib. We observed no toxic effects of Foretinib on normal, non-neoplastic mouse brain, and the ongoing phase II human clinical trials will help elucidate the effects more clearly.

TAM family members are known to crosstalk with other receptors. This study did not examine the cross talk of the TAM family of receptors as they potentially heterodimerize with each other or with RTKs outside the family, but our data illustrates MerTK is inhibited at even the lowest concentrations studied and that Axl is inhibited starting at the moderate doses, as are the common downstream pathways. It is likely that any crosstalk signaling is at the least inhibited similarly. cMet is the RTK that is most closely related to the TAM family and is the described target of Foretinib, and it has been previously shown that Foretinib inhibits cMet and Axl at similar concentrations [38]. Our data demonstrate that inhibition of cMet activation doesn't occur until moderate to high doses of Foretinib, well beyond the concentration needed for inhibition of MerTK, indicating a likely higher affinity for the kinase domain of MerTK. Another study using LY2801653, a small molecule inhibitor closely related to Foretinib, showed that MerTK is inhibited at a 50-fold lower concentration than cMet [39]. Additionally, in the case of the pediatric SF188 cell line, there is little to no activation of cMet at baseline (Figure 1B), suggesting that the downstream signaling changes and phenotypes illustrated in that line are due in large part to inhibition of the TAM family RTKs, and most specifically MerTK.

We investigated the effect of TAM inhibition with Foretinib on both short-term survival of glioblastoma cells using an MTT assay and long-term proliferation in a soft agar colony growth assay and discovered that Foretinib inhibited tumor cell survival in both settings. The MTT assay specifically measures cellular metabolic activity after a short treatment period, and indicates the cells capacity to survive TAM inhibition. With even the single and brief Foretinib treatment cellular survival was greatly reduced, likely through the well described decrease in anti-apoptotic signaling that follows TAM inhibition, which is also evidenced by our data of diminished PI3K and MAPK activation as well as PARP cleavage. We also showed that PARP is cleaved with increasing levels of Foretinib treatment under fed conditions suggesting that Foretinib alone, even without an additional stressor such as starvation, is able to initiate the apoptosis pathway. Non-adherent growth of solid tumors in soft agar has been successfully used as a measure of malignant growth potential, and we used this to further delineate the long-term effects of TAM inhibition with Foretinib. Foretinib treatment greatly diminished long-term colony formation, and there is considerable reduction at the very lowest dose of 100 nM, suggesting the substantial role of MerTK in the malignant growth potential of glioblastoma, as at this low concentration it was the only RTK effectively inhibited among those we have tested.

TAM family receptors act through various kinase pathways that translate into changes in the cytoskeleton and ultimately changes in migration and invasion. Previous investigations have shown that a dominant negative form of Axl resulted in less motility [40] and shRNA against MerTK reduced cellular migration via altered FAK signaling and total RhoA GTPase [21]. We found that Foretinib alone had no effect on total RhoA GTPase, but it remains unclear if it inhibits activation. Additionally, shRNA against MerTK was shown to decrease tumor cell invasion through actomyosin contractility [41]. Our data confirm these findings with Foretinib inhibition of MerTK and, to a lesser extent, Axl and Tyro3, results in attenuated downstream signaling of Akt (2A & C) and FAK (preliminary data). Other targets that are involved in the migratory phenotype in glioblastoma include integrin α3, but it remains to be seen if it is a downstream target of Foretinib [42]. Foretinib interfered with functional effects of MerTK and Axl activation in migration and invasion assays, suggesting its potential as a therapy for GBM, especially as additional downstream targets are elucidated. Prevention of migration and invasion with TAM RTK inhibition in this tumor type is critical as the mainstay of effective therapy is likely to continue to be local control with surgery and radiotherapy.

Temozolomide (TMZ) has been in use to treat glioblastoma for over a decade, and is an alkylating agent that methylates DNA and triggers apoptosis. The promoter methylation status of the MGMT gene dictates the efficacy of this treatment because it is a methylation repair enzyme. In glioblastoma tumors that are sensitive to TMZ, it does reliably extend survival from 12 months to 15 months. In previous work we have shown that inhibition of MerTK and Axl with shRNA improves chemosensitivity to several standard therapies including temozolomide, and here we reiterate that TAM inhibition with Foretinib work effectively together utilizing two cell lines that are described as TMZ sensitive, U251 and A172, and one considered resistant, SF188. Future studies will need to explore other translational TAM inhibition strategies with a variety of cytotoxic chemo- and radio-therapies to determine best sequence and combination of therapies.

Our data illustrate that TAM inhibition either with Foretinib or with genetic knockdown showed robust anti-tumor activity in multiple models of disease. In our experience, U251 cells injected into the subcutaneous flank of nude mice typically smolder for a period of 8-10 weeks before appreciable tumor growth begins; this model mimics a minimal residual disease (MRD) state that might be found in the clinical setting following surgical resection and radiation therapy. Foretinib treatment initiated at 8 weeks post-injection (Foretinib MRD) substantially hindered tumor growth; tumors remained significantly smaller compared to vehicle-treated control until Foretinib treatment was discontinued, at which time tumors showed a quick upturn in growth rate, reaching tumor endpoint size rather rapidly, indicating the potential for ongoing malignant growth that is very effectively diminished by TAM RTK inhibition with Foretinib. The other treatment arm, where Foretinib was delayed until the animal had a rapidly growing established tumor (Foretinib ET, greater than 125 mm^3^), is a model of glioblastoma as it typically presents at diagnosis and relapse; this therapeutic arm also led to considerable reduction of tumor size and control of tumor growth.

Previous reports have established the importance of Axl in human and xenograft glioma tumor growth [13, 40], although those reports did not explore the presence or relative inhibition of MerTK. Our work now demonstrates that MerTK plays an equally critical role. We confirmed the essential role of MerTK with subcutaneous and intracranial models of glioma utilizing lines that had genetic inhibition of MerTK, this allows for both exclusive targeting as well as more thorough inhibition of signaling. The specific and concentrated inhibition of MerTK resulted in profoundly inhibited tumor development, both in the flank and the intracranial orthotopic models. Foretinib therapy provides a unique inhibition of both Axl and MerTK, and future testing will test the relative roles of each TAM member individually and the effects of combination therapy as well as therapeutic penetration through the blood brain barrier.

In conclusion, we have shown that MerTK is essential for glioblastoma growth. Foretinib, an RTK inhibitor that targets TAM family members, with highest efficacy towards MerTK, effectively inhibited proliferation, survival, migration and invasion of human glioblastoma cells *in vitro* by blocking phosphorylation of the TAM kinase motif. TAM RTK inhibition significantly interfered with tumor growth and, importantly, increased median overall survival in both a subcutaneous xenograft and intracranial orthotopic mouse model. By targeting the most characteristic survival and invasive properties of glioblastoma, we assert that TAM inhibition with agents such as Foretinib has the potential to be an important and novel addition to glioblastoma treatment options.

## MATERIALS AND METHODS

### Cell lines and reagents

The U251 and A172 cell lines were obtained from American Type Culture Collection (Manassas, VA, U.S.A.) while the SF188 cell line was obtained from the UCSF Brain Tumor Bank; all were maintained in DMEM + 10% fetal bovine serum per culture guidelines, unless otherwise indicated. Cell lines were authenticated by DNA finger-printing bi-annually through the CU Cancer Center DNA Sequencing and Analysis Core Facility [43]. All antibodies were obtained from Cell Signaling Technology (Danvers, MA, U.S.A.) unless otherwise noted. GSK1363089G (Foretinib) was kindly provided by GlaxoSmithKline (Brentford, Middlesex, UK) via MTA. Stock solutions were prepared in dimethyl sulfoxide (DMSO aka vehicle) and stored in aliquots at −20 °C. All reagents were obtained from Life Technologies, unless otherwise noted.

### Production of shRNA clones

Lentiviral vectors (pLKO.1) containing shRNA sequences targeting MerTK (shMerTK, oligo ID: TRCN0000000862) or non-silencing control green fluorescent protein (GFP; shGFP) were obtained from Open Biosystems. Replication incompetent viral particles were generated using the 293FT cell line and the third-generation packaging system (two packaging plasmids and one envelope plasmid) developed by the laboratory of Dr. Didier Trono. Puromycin-resistant colonies were typically observed on days 9 to 13. Stable, clonal lines were developed from heterogenous MerTK shRNA knockdown cell populations by single-cell flow cytometry sorting for low MerTK expression.

### Immunoprecipitation and immunoblotting of phospho-TAM family members

Cultured cells were treated with Foretinib or vehicle for 1 h. Freshly made pervanadate (0.12 mM Na_3_VO_4_ in 0.0002% H_2_O_2_) was added to samples where indicated as a phosphate stabilizer for 5 min before collecting protein lysate. Lysis buffer (50 mM HEPES, pH 7.5, 150 nM NaCl, 10 mM EDTA, 10% glycerol, 1% Triton X-100, 1 mM Na_3_VO_4_, 0.1 mM Na_2_MoO_4_) with protease inhibitor (Complete Mini, Roche, Mannheim, Germany) was added to each well, rocked on ice for 15 min, and then scraped. The lysate was divided and incubated with the appropriate antibody (MerTK: MAB8912, R&D Systems, Minneapolis, MN; Axl: R&D Systems AF154; Tyro3: Epitomics EPR4308) and Protein G-sepharose beads 4B with rotisserie rotation overnight. Immune complexes were washed thoroughly and heated to 95 °C for 5 min in Laemmli Sample Buffer (62.5 mM Tris-HCl pH 6.8, 25% glycerol, 5% β-Mercaptoethanol, 2% SDS and 0.01% bromophenol blue). Samples were resolved using SDS-PAGE and transferred to nitrocellulose using the iBlot Dry Blotting System (Invitrogen, Carlsbad, CA). Nitrocellulose membranes were blotted with anti-MerTK (ab52968, Abcam, Cambridge, MA) or anti-Tyro3 (Epitomics EPR4308) or anti-Axl (R&D Systems AF154).

### Immunoblotting of downstream kinases and apoptotic proteins

Protein lysates were prepared as described without pervanadate. Total protein concentrations were determined by the Protein 660 nm Assay (Thermo Fisher Scientific, Rockford, IL). Samples were prepared for immunoblotting as described. Antibodies were purchased from Cell Signaling Technology (Boston, MA), unless otherwise indicated. Antibodies used were: Phospho-Met (Tyr1003) (13D11) Rabbit mAb #313, total-Met Rabbit mAb #4560, phospho-Akt (Ser473) (D9E) XP® Rabbit mAb #4060, total-Akt #9272S, Phospho-p44/42 MAPK (Erk1/2) (Thr202/Tyr204) (D13.14.4E) XP® Rabbit mAb #4370, p44/42 MAPK (Erk1/2) Antibody #9102, PARP #9542S, and α-Tubulin (11H10) Rabbit mAb #2125.

### xCELLigence migration assay

The migration assays were conducted as previously described [21, 44]. Briefly, adherent cells at 70% confluency were collected using 0.02% EDTA and resuspended at a concentration of 3 x 10^4^ cells/50 μL. Cells were plated in triplicate with media in the CIM-plate 16 (ACEA Biosciences, San Diego, CA) and allowed to adhere to the plates free of therapeutics for 1 h. Cells were treated with either vehicle or a serial dilution of Foretinib in DMEM + 10% FBS (final concentrations of 900nM, 300nM, and 100nM). Migration was quantified every 10 min for 20 h. Three independent experiments were performed.

### Neurosphere 3D collagen invasion assay

1 x 10^4^ cells were cultured in Neurobasal Medium A supplemented with B27, 0.5 mM L-glutamine, 20 ng/mL hEGF (Sigma-Aldrich, St. Louis, MO), 20 ng/mL bFGF (Sigma-Aldrich) in ultra-low adherent 24-well plates (Corning Life Sciences, Corning, NY) for 24 h to allow formation of neurospheres [35]. Neurospheres of 75-150 um were resuspended in type I collagen (2.17 mg/mL)/DMEM + 10% FBS and appropriate treatments and plated in a 96-well plate pre-coated with type I collagen (Advanced BioMatrix, San Diego, CA). Neurospheres were imaged immediately (Time 0) with an Olympus CKX41 fitted with a Qicam Fast 1394 camera and then again at 15 and 24 h. Absolute maximal radius of each neurosphere was measured using ImageJ software and this distance was used to determine the relative change in radius. Three independent experiments were performed.

### Soft agar colony formation

Six-well plates were layered with 0.5% agar in 1.5 mL of DMEM + 10% FBS, followed by plating of cell lines suspended in 0.35% agar in 1.5 mL DMEM + 10% FBS. U251 cells were plated at 3 x 10^4^ cells per well, while A172 and SF188 cells were plated at 1 x 10^5^ cell per well. Treatments (DMSO or Foretinib) were added to triplicate wells. Cells were incubated for 3 weeks at 37 °C and 5% CO_2_, with replacement of media and treatment twice weekly. The cells were stained with nitrotetrazolium blue (1 mg/mL) 48 h prior to counting and colonies were scored electronically with an automated colony counter, GelCount (Oxford Optronix, Oxford, UK). Three independent experiments were performed.

### Survival and proliferation using xCELLigence

An xCELLigence E-plate 16 containing only 100 μL of DMEM + 10% FBS was incubated for 30 min at room temperature and scanned on the xCELLigence RTCA. A serial dilution of Foretinib in DMEM +10% FBS and vehicle control solution was prepared as previously described. Adherent cells at approximately 70% confluency were collected using 0.02% EDTA and resuspended at a concentration of 1 x10^4^/ 100 μL, plated in triplicate, and incubated at room temperature for 30 min to allow for cell adherence. Cell index, which represents total cell number, was measured every 15 min over the length of the experiments. Three independent experiments were performed.

### MTT assay

Cells were seeded on 96-well cell culture plates and allowed to recover overnight. Media was aspirated and replaced with media containing treatments (Foretinib, temozolomide, vehicle, or serum free media). Two days after treatment, MTT reagent (3-(4, 5-dimethylthiazol-2-yl)-2, 5-diphenyl tetrazolium bromide) was added to each well and incubated for 4 h. Next, solubilization solution (10% SDS, 0.01 M HCl) was added and incubated overnight. The plates were read at absorbance 570 nm and 690 nm for normalization purposes. Four independent experiments were performed.

### Xenograft mouse models

Athymic nude in a *Balbc* background (*Foxn1^nu^*) mice were purchased from Harlan Laboratories (Indianapolis, IN). Animal care and experimental procedures were conducted in accordance with the guidelines of the University of Colorado Center for Comparative Medicine and the University of Colorado Institutional Animal Care and Use Committee. All experiments included several (n=3-5) mice in each control and experimental group and each of the experiments was independently repeated on two occasions separated by at least a month. Mice were maintained on 0.3 mg/ml amoxicillin and 0.07 mg/ml clavulonic acid (Clavamox) in drinking water (Pfizer Animal Health, New York, NY). For the subcutaneous mouse models, five- to seven-week old nude mice were injected subcutaneously with 3 x 10^6^ U251 cells suspended in a total volume of 150 μl serum-free RPMI (HyClone Laboratories, Inc., Logan, UT). Tumors were measured by caliper twice weekly starting one week post injection (p.i.); volume was estimated by the calculation V = LW^2^/2. Mice in the Foretinib treatment evaluation were randomly assigned to either control or one of two experimental treatment regimens and were treated every other day by oral gavage with vehicle [VEH: 1% hydroxypropylmethylcellulose (Ashland Specialty Ingredients, Wilmington, DE) and 0.2% sodium lauryl sulfate (Sigma Aldrich Corp., St. Louis, MO) in di H_2_0] or with Foretinib 30 mg/kg suspended in VEH. The first treatment regimen cohort began therapy at 8 weeks p.i. when tumors were similar to minimal residual disease state (Foretinib MRD). The second treatment regimen group began when the tumor volume reached at least 125 mm^3^, as this approximated when the tumor was well-established (ET) and began rapid logarithmic growth, similar to what is seen typically at initial clinical diagnosis or recurrence. A discontinuation phase was also analyzed, following 8 weeks of Foretinib therapy (16 weeks p.i.), mice were transitioned to VEH. Tumor volume was monitored until mouse reached defined morbidity endpoint, tumor was 2500 mm^3^ or greater for two measurements, or 30 weeks p.i.. Two tumors (vehicle and foretinib treated) were resected and immediately homogenized in lysis buffer with protease inhibitor with and without freshly prepared pervanadate. The protein samples were prepared for gel electrophoresis and immunoblotting. The nitrocellulose membrane was incubated with the phospho MerTK antibody (ab52968) overnight, then stripped and reprobed for total MerTK (MAB8912) the following day. For the orthotopic mouse model of human glioblastoma that was developed to study tumor growth in the context of brain microenvironment, a small volume of U251 shMerTK or control parental cells (4x10^4^) were suspended in serum-free DMEM and injected into the cerebral cortex at 2.5 mm lateral (right) and 1.5 mm anterior to the bregma, at a depth of 3.5 mm below the skull surface of athymic nude mice (6 to 12 weeks old) that are anesthetized and immobilized. A microultrapump injected the cell suspension at a very slow rate (400 to 600 nl/min) to minimize leakage into surrounding tissues. Tumor growth was monitored by magnetic resonance imaging (MRI) with and without gadolinium injection approximately every 3 weeks. Tumor volumes were measured by Paravision Preclinical MRI Software (Bruker, Billerica, MA).

### Statistical Analysis

Appropriate statistical analyses were performed as indicated using Prism 5 (Version 5.04, Graphpad Software, Inc., La Jolla, CA, USA).

## SUPPLEMENTARY FIGURE


