# Supplementary material for: MerTK inhibition is a novel therapeutic approach for glioblastoma multiforme
Source: Oncotarget. 2014 Mar 12;5(5):1338–51. doi: 10.18632/oncotarget.1793 (PMC4012720; doi:10.18632/oncotarget.1793)
Supplement: Supplementary file 1 [file oncotarget-05-1338-s001.pdf]

MerTK inhibition is a novel therapeutic approach for glioblastoma multiforme – Knubel et al

A.

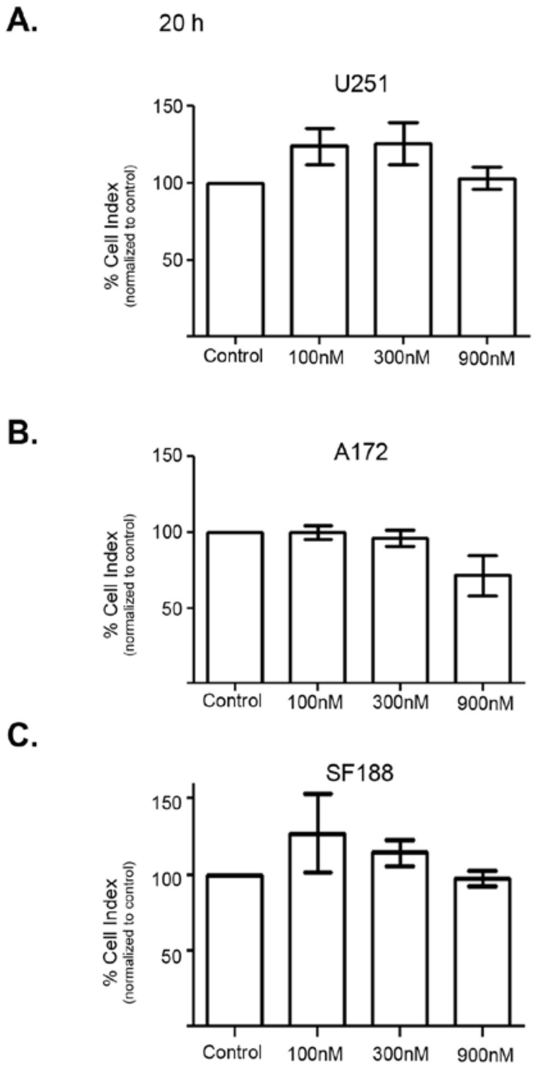

Supplemental Figure 1. <u>Cellular survival and proliferation does not account for the effect of Foretinib on migration.</u> a. U251, b. A172, and c. SF188 cells were plated in triplicate in xCELLigence E-Plates in a parallel manner to the migration experiments. Cells were treated with vehicle only (Control) or Foretinib at the indicated doses and cell number was measured as electrical impedence at 20 hrs. The experiment was independently repeated at three times and cell index means and standard deviations were calculated and normalized to vehicle control. A repeated measurements ANOVA with a Dunnet's multiple comparison was done to compare treatment to control and no significant difference was found between any of the treatments.
